# Stress in migraine: personality-dependent vulnerability, life events, and gender are of significance

**DOI:** 10.3109/03009734.2011.573883

**Published:** 2011-06-29

**Authors:** Kerstin Hedborg, Ulla Maria Anderberg, Carin Muhr

**Affiliations:** ^1^Faculty of Health and Occupational Studies, Department of Health and Caring Sciences, University of Gävle, Sweden; ^2^Department of Medical Sciences, Uppsala University, Sweden; ^3^Department of Public Health and Caring Sciences, Social Medicine, Uppsala University, Sweden; ^4^Department of Neuroscience, Psychiatry, Uppsala University, Sweden

**Keywords:** Gender, life event, migraine, personality, stress

## Abstract

**Background and aim:**

The individual's experiences of stress as well as constitutional factors, including high neuroticism and female gender, are known determinants for migraine. The present aim was to further elucidate factors of personality and stress, including life events, in relation to gender in migraine.

**Methods:**

A cross-sectional study was performed on 150 persons, 106 women and 44 men, suffering from at least two migraine attacks a month. All obtained a doctor-defined migraine diagnosis based on a structured face-to-face interview concerning their health situation and current and prior stress. All of them also answered validated questionnaires regarding personality traits (SSP), life events, and perceived ongoing stress.

**Results:**

The personality trait inventory showed high mean scores for stress susceptibility and low mean scores for aggressiveness and adventure seeking, both for women and for men, as well as high mean scores for psychic and somatic anxiety in women. Stress susceptibility, the overall most deviant trait, correlated strikingly with current level of stress in both sexes. In women, stress susceptibility also correlated strongly with experiences of negative life events. Tension-type headache, anxiety, and depression were approximately twice as prevalent in women compared to men.

**Conclusions:**

The present study confirms previous research, showing that stress is an important factor in migraine. Stress susceptibility, life events, and concomitant psychosomatic illnesses should be considered important when evaluating individuals with migraine, and gender aspects need to be taken into account.

## Introduction

Migraine is one of the most common neurological disorders (1,2). Both constitutional and environmental factors have been shown to be important for its clinical manifestations (3). Several studies have identified stress as a major trigger of migraine attacks (4–10). The stress response involves activation of the sympathetic nervous system and the hypothalamic–pituitary–adrenal axis, and it is generally associated with a subjective feeling of external or internal threat or demand (11). The intensity of this reaction is dependent on the duration, frequency, and severity of the stressors as well as the current health condition of the affected individual. Some stressors are a genuine part of human physiology, but many are largely contextually defined and dependent on culture, life situation, previous experiences, and personality factors (12–15). However, it has been shown that psychological stress plays an important role not only before the onset of migraine (9,10), but also in the maintenance of the disorder, the frequency of attacks (16,17), as well as the change from episodic to chronic migraine (18). It has also been shown that migraine is associated with several other health conditions of both somatic and psychological character (14,19,20). Furthermore, it is well recognized that there is a strong influence of the female sex hormones in this disorder (21).

The importance of personality traits in migraine has been widely debated. Claims have been made that migraineurs display increased neuroticism and anxiety and are anti-aggressive, but complex interactions regarding personality traits need to be considered (22–24). It may be that some of these personality traits also could be an effect of serious and insufficiently processed life events, as it is shown that migraine patients may have a history of maltreatment, especially during childhood (19,25,26). Furthermore, migraineurs display increased psychiatric co-morbidity such as post-traumatic stress disorders, anxiety disorders, and depression (15,19), which may be the result of constitutional as well as of environmental factors. Patients with migraine also have an increased co-morbidity with somatic disorders, especially chronic pain disorders such as fibromyalgia, arthritis, and orofacial pain (20,27) as well as an increased frequency of menorrhagia and endometriosis (28).

Women and men display differences in the prevalence of many disorders that could be considered to be stress-related (12), including migraine (1,2). Gender differences in prevalence and intensity of migraine may also be effects of gender socialization (29), as it is well known that the gender roles of women and men, both professionally and in private life, are strong determinants of their life situation (30,31).

In view of the complexity of the roles of personality, stress, life events, and gender in individuals with migraine, the aim of the present study was to examine and describe these aspects in a cross-sectional study of 150 migraineurs.

## Materials and methods

### Study population

The study population comprised 150 consecutively enrolled adults (18 years of age and above), 106 women and 44 men, with moderate to severe migraine, defined as two or more migraine attacks a month. The subjects were recruited through advertisements in the local daily newspaper. All participants were interviewed by K.H. and C.M. A medical history was recorded regarding headache characteristics, stress as a trigger for migraine, concomitant illnesses, and general well-being, followed by a neurological examination performed by one of the researchers, C.M., a specialist in neurology, thus ensuring a uniform mode of data collection. The migraine diagnosis was confirmed on the basis of the International Classification of Headache Disorders (32). The study was approved by the Regional Research Ethics Committee, and informed consent was obtained. Eighty-three migraneurs from the same population participated in a further study applying a multimodal behavioral treatment program (33). 

### Questionnaires

In connection with the interview, all participants answered questionnaires, which are described below, on a computer. Complete responses were obtained from all participants. The time required to answer these questionnaires was approximately one hour. All answers were directly transferred into the Statistical Package for the Social Sciences 18.0 (SPSS) program for further analysis.

### The Swedish universities scales of personality (SSP)

SSP was used to assess personality traits (34). This instrument is a revised version of the Karolinska Scales of Personality inventory, which was developed to identify stable personality traits of importance for psychological vulnerability (35).The SSP inventory comprises 91 items divided into 13 traits: 1) *Somatic trait anxiety* (autonomic disturbances, restlessness, tension); 2) *Psychic trait anxiety* (worry, anticipation, lack of self-confidence), 3) *Stress susceptibility* (easily fatigued, feeling of unease when urged to speed up); 4) *Lack of assertiveness* (lack of ability to speak up and to be self-assertive in social situations); 5) *Impulsiveness* (acting on the spur of the moment, non-planning, impulsive); 6) *Adventure seeking* (avoiding routine, need for change and action); 7) *Detachment* (avoiding involvement in others, withdrawn, schizoid); 8) *Social desirability* (socially conforming, friendly, helpful); 9) *Embitterment* (unsatisfied, blaming and envying others); 10) *Trait irritability* (irritability, lack of patience); 11) *Mistrust* (suspiciousness, distrust of people's motives); 12) *Verbal trait aggression* (getting into arguments, berating people when annoyed); and 13) *Physical trait aggression* (getting into fights, starting fights, hitting back). Each item is a statement, to which the participant responds by choosing an alternative on a four-point scale: ‘does not apply at all’, ‘applies to a certain extent’, ‘applies fairly well’, and ‘applies completely’. The SSP inventory results were standardized to normative data. The normative data were drawn from a random sample of the Swedish population, with the normative average for each trait defined as 50 and with a standard deviation of 10. Factor analysis for the instrument (34) showed one factor consisting mainly of traits 1–4 and 9, which was assessed as traits of neuroticism, another factor consisting of traits 8 and 10–13 was assessed as forms of aggressiveness, and a third factor consisting of traits 5–7 was assessed as an extroversion factor.

### The department of environmental stress disorders questionnaire (CEOS inventory) on demographics, current stress, and concomitant illnesses

For evaluation of current stress, a questionnaire containing 75 questions, developed for clinical research at the Department of Environmental Stress Disorders (CEOS), Uppsala University, Sweden, was used (36–38). Forty-three items regarding stress-related psycho-social factors and stress coping, general health, and sleep were measured on a visual analog scale (VAS) graded from 0 to 100 (0 = low/no stress; 100 = maximal stress; Appendix); 38 of these 43 items refer to experiences of stress during the past month and 5 refer to the past or next year. The remaining 32 items concerned demographic data and concomitant illnesses. Items concerning the latter category were: Are you presently treated or have you previously been investigated or treated for: anxiety and depression, gastrointestinal diseases, allergies, cardiovascular diseases? Answering options were: never, yes previously, yes presently. In the further analyses the answers were dichotomized into: never or yes (previously and/or presently). Subjects were also asked if they suffered from tension-type headache.

To identify groups of the 43 stress-related items, a factor analysis with Varimax rotation was conducted on the current responses to the 43 items answered on a VAS scale. The eight factors with the highest eigenvalues were chosen to represent a measurement of current stress, all of which had eigenvalues above 1.0. These factors covered 29 of the 43 stress-related items (Appendix). Our designations of these factors were as follows and are listed in order of magnitude of their eigenvalues: Factor 1: *Feeling of meaningfulness regarding your home situation and your social situation*; Factor 2: *Mental symptoms*; Factor 3: *Subjective level of stress*; Factor 4: *Quality of sleep*; Factor 5: *General health*; Factor 6: *Difficulties with concentration and memory*; Factor 7: *Ability to prioritize recovery from stress*; Factor 8: *Ability to cope with stress.* The reliability of these eight components was tested using the Cronbach method, which yielded alpha values between 0.730 and 0.897 (Appendix).

### Life events inventory

As a measure of stress in the long-term, life events were evaluated. These were separated into events that had occurred during childhood/adolescence and during adulthood. The respective periods were defined by an age limit of 18 years. The instrument used (39,40) consisted of 11 childhood/adolescence life event categories and 21 adulthood categories, listed in Table IV. The participants rated the impact of the events in the respective categories into three grades: ‘strongly negative’, ‘notably negative’, and ‘hardly negative at all’. For the last seven adulthood life event categories listed in Table IV, a ‘positive’ response alternative was also provided. In the further analyses, the data were grouped into either event categories in which the participants had had experiences they rated as ‘strongly negative’ or into ‘any’ type of negative event, including all categories in which the rating was any of the following: ‘strongly negative’, ‘notably negative’, and ‘hardly negative at all’.

### Statistical methods

Differences in proportions were analyzed using the chi-square test. Mean differences were examined using either the *t*-test or the Mann–Whitney test, depending on the distribution and sample size of the variables. Covariation was analyzed using correlation analyses, and the result was expressed as the Spearman's rho coefficient. Factor analysis was performed by means of principal components analysis with Varimax rotation. The number of latent variables was determined by using eigenvalues above 1.0 and scree plot analysis. Cronbach's alpha was used as a measure of the reliability of each component. All analyses were performed with SPSS 18.0 software. The significance level was uniformly set at 0.05, two-tailed test.

## Results

### Demographics

**Figure 1.**
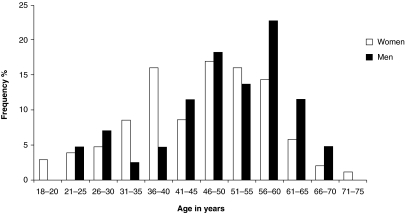
Age distribution at the time of inclusion of the studied women (*n* = 106) and men (*n* = 44) with migraine.

The distribution of participant age at inclusion is shown in 5-year intervals in Figure 1. Additional demographic data on age, body mass index, and education are presented in Table I. The data were separated by gender.

**Table I.** Demographic profiles of women and men with migraine. Statistical comparisons were based on Mann–Whitney test and chi-square analysis.

			Women versus Men	
	Women (*n* = 106)	Men (*n* = 44)	*Z*/chi-square	*P* value
Age, years:				
Range; mean (SD)	18–71; 45.6 (11.9)	25–68; 50.0 (11.5)	*Z*; 2.2	0.026
Body mass index, kg/m^2^:				
Range; mean (SD)	18.3–41.0; 24.7 (4.2)	21.2–36.3; 25.6 (3.5)	*Z*; 1.7	0.089
Education:	% of women	% of men		
College/postgraduate studies	55.7	52.3		
Upper secondary school	34.0	31.8	χ^2^; 0.90	0.637
Nine-year compulsory school	10.4	15.9		

### Clinical migraine characteristics

Migraine frequency, time since onset and age at onset of migraine, presence of aura and associated symptoms, pain degree, chronicity of symptoms, and menstrual correlation are presented in Table II. All data were separated by gender, and sex differences were analyzed.

**Table II.** Clinical migraine characteristics and concomitant illnesses in women and men. Statistical comparisons were based on Mann–Whitney test and chi-square analysis.

			Women versus Men	
	Women (*n* = 106)	Men (*n* = 44)	*Z*/chi-square	*P* value
Migraine, attacks/month:				
Range; mean (SD)	2–20; 4.6 (3.7)	2–15; 4.8 (3.9)	*Z*; 0.3	0.812
Time since onset, years:				
Range; mean (SD)	1–64; 23.1 (13.1)	5–59; 28.1 (15.7)	*Z*; 2.0	0.048
Age at onset, years:	% of women	% of men		
≤15	44.3	56.8	χ^2^; 1.94	0.164
16–30	41.5	20.5	χ^2^; 6.03	0.014
31–40	8.5	13.6	χ^2^; 0.92	0.339
41–50	5.7	9.1	χ^2^; 0.58	0.443
Migraine with aura	35.8	43.2	χ^2^; 0.71	0.400
Associated symptoms:				
Nausea	81.1	59.1	χ^2^; 7.98	0.005
Photophobia	84.0	77.3	χ^2^; 0.94	0.332
Phonophobia	75.5	63.6	χ^2^; 2.16	0.142
Osmophobia	50.9	20.5	χ^2^; 11.86	<0.001
Pain degree in migraine:				
Severe	93.4	84.1	χ^2^; 3.18	0.074
Chronic migraine^a^	3.8	4.5	χ^2^; 0.05	0.826
Menstrual related migraine	75.5	–		–
Concomitant illnesses:				
Gastrointestinal diseases	48.1	40.9	χ^2^; 0.65	0.420
Tension-type headache	46.2	20.5	χ^2^; 8.71	0.003
Allergies	43.4	38.6	χ^2^; 0.29	0.591
Anxiety and mental depression	42.5	20.5	χ^2^; 6.53	0.011
Cardiovascular diseases	13.2	18.2	χ^2^; 0.62	0.433

^a^15 attacks/month or more.

### Gender differences in associated symptoms and concomitant disorders

Most notable gender differences, with up to double the frequency in women compared to men, were: associated nausea and osmophobia, tension-type headache, anxiety, and depression. Gender differences were seen also in age at migraine onset (Table II). The perceived role of stress as a trigger for migraine attacks was measured on a VAS scale 0–100 millimeters (0 = no impact; 100 = total impact). Women scored 75.6 on average (SD 24.1) versus men's average score of 58.5 (SD 29.7); *P* < 0.001 (*Z* = 3.7), Mann–Whitney test.

### Personality traits

Personality traits were analyzed by gender and are shown in Table III. Mean scores for personality traits that deviated significantly from normative means in both women and men were: high stress susceptibility, low verbal trait aggression, low physical trait aggression, and low adventure seeking. Women with migraine, but not men, also displayed significantly higher values than the normative reference for somatic trait anxiety and psychic trait anxiety. The most deviant personality trait for the entire study group was high stress susceptibility (data not shown).

**Table III.** Personality trait scores in women and men with migraine. Mean scores for the Swedish universities Scales of Personality inventory (SSP), compared to normative data (mean = 50; SD = 10), separated by gender. Statistical comparisons were based on one-sample *t*-test.

	Women (*n* = 106)		Men (*n* = 44)	
	Mean (SD)	*P* value versus controls^a^	Mean (SD)	*P* value versus controls^a^
Stress susceptibility	55.3 (10.2)	<0.001	53.5 (10.9)	0.037
Somatic trait anxiety	53.7 (9.3)	<0.001	51.5 (6.9)	0.144
Verbal trait aggression	47.0 (8.6)	<0.001	46.9 (8.7)	0.025
Physical trait aggression	47.5 (8.9)	0.004	45.9 (6.7)	<0.001
Adventure seeking	47.7 (8.5)	0.007	47.0 (9.7)	0.046
Psychic trait anxiety	51.9 (9.9)	0.046	52.1 (11.3)	0.224
Embitterment	51.6 (9.9)	0.104	50.1 (7.4)	0.954
Lack of assertiveness	51.5 (10.8)	0.154	51.9 (10.7)	0.257
Detachment	48.7 (9.6)	0.162	47.6 (9.8)	0.113
Impulsiveness	48.8 (11.1)	0.254	49.4 (9.3)	0.695
Trait irritability	51.1 (11.7)	0.316	48.5 (8.7)	0.266
Social desirability	49.6 (8.3)	0.632	51.4 (9.9)	0.350
Mistrust	48.2 (10.1)	0.062	47.7 (8.5)	0.081

^a^Controls were provided in the instrument (see Materials and methods section).

A factor analysis of our data on personality traits yielded a neuroticism factor very similar to that of the normative data of the SSP instrument (34), with somatic trait anxiety (factor loading 0.56), psychic trait anxiety (factor loading 0.88), stress susceptibility (factor loading 0.79), lack of assertiveness (factor loading 0.81), and embitterment (factor loading 0.66) loaded on the neuroticism factor (eigenvalue 4.25). The reliability of these five scales was tested using the Cronbach method, which yielded an alpha value of 0.823.

### Life events

**Figure 2.**
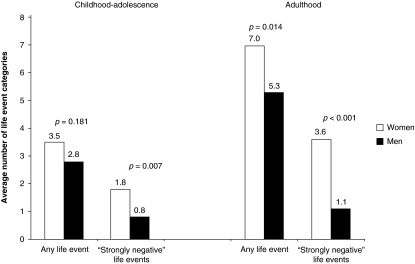
Life events in women and men with migraine: the *y*-axis shows the average numbers of life event categories reported, separated by ‘any’ negative event and events with a ‘strongly negative’ influence, as well as by gender and period in life in which they occurred. The Mann–Whitney test was used for statistical analysis of gender differences in average numbers.

Details of event categories rated as having had a ‘strongly negative’ impact are presented by gender in Table IV, and Figure 2 summarizes data on life event categories according to the grouping into ‘any’ negative event and ‘strongly negative’ events, separated by gender and age period during which they occurred. Women reported life events in significantly more event categories and also significantly more event categories in which events had had a “strongly negative” impact, both during childhood/adolescence and during adulthood (Table IV; Figure 2).

**Table IV.** Life events categories with a strongly negative impact in women and men with migraine. Statistical comparisons were based on chi-square analysis.

	Women (*n* = 106)	Men (*n* = 44)	Women versus Men	
	% of Women	% of Men	Chi-square	*P* value
**Childhood/adolescence:**				
Conflict with parents	23.6	11.4	2.9	0.088
Conflict with close relative or friend	18.9	9.1	2.2	0.137
Support for close relative (e.g. due to drug abuse, old age, disability)	12.3	0	5.9	0.015
Physical or psychological abuse	15.1	11.4	0.4	0.549
Neglect	14.2	4.5	2.9	0.091
Sexual assault	9.4	4.5	1.0	0.315
Own disease or accident	14.2	2.3	4.6	0.032
Disease or accident in close relative	19.8	9.1	2.6	0.109
Death of parent	17.9	13.6	0.4	0.521
Death of close relative or friend	22.6	13.6	1.6	0.209
Bullying	15.1	4.5	3.3	0.070
Any experience of strongly negative life event(s)	60.4	38.6	5.9	0.015
No experience of a strongly negative life event	39.6	61.4		
**Adulthood:**				
Conflict with partner	36.8	22.7	2.8	0.094
Conflict with children, close relative or close friend	28.3	18.2	1.7	0.194
Support for close relative or friend (e.g. due to drug abuse, old age, disability)	17.9	6.8	3.1	0.080
Physical or psychological abuse	20.8	6.8	4.3	0.037
Sexual assault	8.5	2.3	1.9	0.165
Own disease or accident	15.1	2.3	5.1	0.024
Disease or accident in close relative	33.0	4.5	13.6	<0.001
Death of partner	2.8	0	1.3	0.260
Death of close relative or friend	41.5	18.2	7.5	0.006
Bullying	18.9	2.3	7.1	0.008
Financial problems	12.3	9.1	0.3	0.577
Conflicts at work	21.7	0	11.3	0.001
Lack of control of work situation	20.8	2.3	8.2	0.004
‘Other’ important negative events	25.5	6.8	6.7	0.009
Marital separation	24.5	9.1	4.6	0.031
Marriage	3.8	2.3	0.2	0.640
Move to a new home	7.5	0	3.5	0.061
Birth of child or adoption	0.9	0	0.4	0.518
New job	8.5	0	4.0	0.046
Decreased responsibility at work	2.8	0	1.3	0.260
Increased responsibility at work	2.8	0	1.3	0.260
Any experience of strongly negative life event(s)	79.2	52.3	11.1	0.001
No experience of a strongly negative life event	20.8	47.7		

The life events reported in the present study were also compared to those of a healthy control group (45 women and 30 men) presented in another study from the same time period and geographical region (40). When analyzing these data in relation to age-matched women (*n* = 57) and men (*n* = 24) from our study population, it appears that our migraineurs had experienced significantly more events—about twice as many. The respective ratios (present study versus controls in Marteinsdottir et al. (40)) for women and men were: ‘any’ childhood/adolescence event, women: 1.7 (*P* = 0.006; Mann–Whitney test) and men: 1.9 (*P* = 0.015); ‘strongly negative’ childhood/adolescence events, women: 2.4 (*P* = 0.017) and men: 1.5 (*P* = 0.598); ‘any’ adulthood event, women: 2.0 (*P* < 0.001) and men: 2.6 (*P* < 0.001); ‘strongly negative’ adulthood events, women: 3.4 (*P* < 0.001) and men: 2.2 (*P* = 0.139).

### Correlations between personality, life events, and current stress

A gender-based correlation analysis was performed to discern interdependence between personality and stress as measured by life events or as current stress according to our CEOS instrument. For this purpose we selected the overall most deviant personality trait—stress susceptibility—which was compared to scores for negative life events and to factors of current stress in the present study, respectively: correlation analysis (Spearman) between stress susceptibility scores and number of life events revealed that these parameters were correlated in women, independent of the severity of the life events or the period in life in which they occurred (Table V). In men, a positive correlation was seen between stress susceptibility scores and the overall number of events during adulthood, which was not the case for events occurring during childhood/adolescence or for events rated as ‘strongly negative’ (Table V). With regard to the correlation between scores for stress susceptibility and scores for each of the eight factors of current stress, as defined by our factor analysis of the CEOS inventory results, women displayed significant positive correlations for seven and men for six out of eight of these factors (Table V). In both women and men, this correlation was strongest for the ‘difficulties with concentration and memory’ factor.

**Table V.** Correlations between stress susceptibility scores in women and men with migraine and A: scores for life events; B: scores for current stress. Correlations were analyzed using Spearman's rho coefficient.

		Women (*n* = 106)		Men (*n* = 44)	
		Spearman's rho	*P* value	Spearman's rho	*P* value
A	Categories of life events:				
	Childhood/adolescence				
	‘Any’ event	0.34	<0.001	-0.07	0.674
	‘Strongly negative’ events	0.34	<0.001	0.17	0.263
	Adulthood				
	‘Any’ event	0.35	<0.001	0.30	0.049
	‘Strongly negative’ events	0.33	0.001	0.22	0.147
B	Factors of current stress:				
	Difficulties with concentration and memory	0.50	<0.001	0.62	<0.001
	Mental symptoms	0.48	<0.001	0.49	0.001
	Ability to cope with stress	0.46	<0.001	0.61	<0.001
	Feeling of meaningfulness of home and social situation	0.44	<0.001	0.55	<0.001
	Quality of sleep	0.38	<0.001	0.17	0.171
	Subjective level of stress	0.37	<0.001	0.29	0.290
	General health	0.35	<0.001	0.46	0.002
	Ability to prioritize recovery from stress	0.15	0.147	0.38	0.010

## Discussion

### Migraine and personality

The aim of the present study was to elucidate factors of personality and of past and on-going stress in migraineurs and the gender dependence of these factors. With regard to personality, we found that stress susceptibility was the overall most deviant trait. Women displayed significantly elevated scores also for the anxiety traits of the neuroticism cluster, and both sexes scored significantly lower for traits of aggression and adventure seeking than normative data. These profiles are in concordance with previous findings on personality traits in migraineurs (23,24). Regarding traits of neuroticism in women and men with migraine, Merikangas et al. (41) reported increased neuroticism in both sexes in a longitudinal study on migraine subjects between 19 and 29 years of age. Breslau et al. (42) showed that high neuroticism was associated with a higher incidence of migraine, which was more marked for women. High neuroticism levels were also found by Huber and Henrich (43), who studied women with migraine. The high values on the two neuroticism traits psychic and somatic anxiety for women in this study are in line with other reports where the researchers have found that migraine patients were more alexithymic, anxious, and depressed—especially those making frequent health care visits for their migraine (‘repeaters’) (44). Moreover, in another study the authors found that migraineurs displayed signs of low assertiveness, as evidenced by their avoidance of seeking social support to the same extent as healthy controls when they were experiencing stressful events (45).

Apart from traits of neuroticism, we found low average scores for traits of aggression and for adventure seeking in both sexes in the present study, which is in agreement with early data by Wolff, who suggested that migraineurs repress strong emotions, in particular aggression (24). It is possible that repressing feelings, especially those of anger, may increase the perception of stress, which in turn may affect the course of migraine. This personality-dependent mechanism could work in synergy with high stress susceptibility in causing a stronger impact of stressors in migraineurs.

### The perception of stress

Previous research speaks strongly in favor of the negative influence of stress on migraine (4,5,7,8,10). However, women and men display differences in their susceptibility to stressors (12,29,46). For example, claims have been made that women are more communally oriented and men have higher agentic, instrumental tendencies (46) and that women, therefore, are more reactive to social rejection challenges whereas men react more to achievement challenges (29). These differences, however, may be dependent also on social and cultural factors and may hence change with time (12).

Interestingly, we found that our measurements of current stress were highly correlated with scores for the most deviant personality trait found, stress susceptibility. Intuitively, this appears logical, but we have not found any reference to this correlation in previous personality studies regarding migraine. It has been reported that migraineurs are not exposed to external stress to a greater degree than comparable controls (43), which would indicate that the personality-dependent differences in stress level observed are primarily due to how external demands are perceived. We find the correlation between stress susceptibility and different items of current stress worth discussing, because this correlation was strong in both women and men. In women a strong correlation to life events was also seen. The extent of these correlations further underscores the great importance of stress in migraine. A consequence of the fact that stress susceptibility is strongly correlated to parameters of stress is that either measurement—stress susceptibility or stress—can be used interchangeably in the individual characterization of migraine patients, which is similar to what Huber and Henrich have expressed (43).

### Life events

Regarding life events, obvious gender differences were seen in the present study. Overall, women reported approximately three times more experiences of strongly negative events. When separated by individual event category, higher female rates for such experiences were reported in all 32 categories, reaching statistical significance in 12 of them. These clear gender differences may reflect both differences in life situations for women and men and gender-dependent differences in how the events were perceived. For example, it is possible that life events related to the support of close relatives or friends are more negative for women because they face greater expectations to provide care. On the other hand, it seems reasonable that gender differences in perception of stress could explain why the women more often rated life events concerning their own disease, or the death of a close relative or friend, as having a strong negative influence. It is therefore possible that women are more sensitive to all kinds of stress. This is indicated by several studies showing that women experience more anxiety and depressive disorders, particularly in relation to different kinds of long-lasting perceived stress e.g. in fibromyalgia—another pain disorder that also is found to be related to stress and negative life events (39,47,48). Compared to healthy controls from the study by Marteinsdottir et al. (40), the participants of this study displayed approximately twice as many life events independently of time period in life and of gender. It has also been found previously that migraineurs experience much more negative life events during childhood than healthy persons (26). Furthermore, an independent support for a correlation between life events and migraine was provided by Peterlin et al. (15) in a study on post-traumatic stress disorders in migraine. However, these events were generally of greater magnitude than those reported in the present study.

### Secondary findings regarding gender differences in concomitant anxiety, depression, and tension-type headache

It is well documented that migraine is concomitant with psychiatric disorders such as depression, anxiety, and post-traumatic stress disorder (15,19,49). It is notable that in the present study ongoing or a history of previous anxiety and/or depression were reported twice as frequently by the women as by the men. Again, it is possible that the high incidence of concomitant psychiatric disorders in migraineurs may worsen the migraine when these disorders have not been addressed and treated adequately. Tension-type headache was also considerably more prevalent in women than in men in the current study, which is also known from earlier studies (50,51). These gender differences in concomitant psychiatric/psychosomatic disorders are in line with the other observed gender differences in personality traits and in life events of this study and emphasize the importance of taking gender into account when evaluating migraine patients.

### Limitations

The number of male participants in our study was more limited compared to the number of females, despite special efforts to recruit men. Hence, low statistical power may have led to lack of detection of correlations in the male group as well as to detection of gender differences. Due to our mode of recruitment via advertisements in the local press, selection factors may also have influenced our results. Given its cross-sectional design, the study is descriptive in nature, and thus drawing conclusions about causality is precluded. Another weakness is the lack of a control group. Regarding life events, a healthy, age-matched control group from another study provided a possibility to compare our data, as described. With respect to personality we used normative data provided in the SSP instrument. Our own factor analysis of the SSP results yielded a profile similar to that described in the instrument, strengthening the validity of the instrument in our setting.

### Concluding remarks

The present study confirms previous research showing that stress is an important factor in migraine. It indicates that high stress susceptibility is characteristic for migraine sufferers, and that stress susceptibility correlates with the individual's level of stress. Clear gender differences were found in personality traits and in the number of stressful life events, in particular those rated as strongly negative. Furthermore, anxiety, depression, and tension-type headache were twice as frequent in women. We conclude that stress susceptibility, life events, and concomitant disorders, especially of psychiatric nature, ought to be considered when investigating and treating individuals with migraine.

## Appendix

The CEOS inventory developed at the Department of Environmental Stress Disorders, Uppsala University, Uppsala, Sweden.

**Table:**

It consists of the following 43 items:	Response options were given on a VAS scale for each item, graded from 0 to 100:	
*For each of these statements make a mark on the VAS scale. Choose a point on the scale which applies best to your situation.*	0	100
1. Describe your health status during the past year.^5^	excellent	poor
2. What is your health status right now?^5^	excellent	poor
3. What do you think your health status will be next year?^5^	excellent	poor
4. I have felt great strain during the past year.^3^	do not agree	agree totally
5. I feel great strain right now.^3^	do not agree	agree totally
7. What is your quality of sleep right now?^4^	excellent	poor
8. My life has felt meaningful during the past year.^1^	agree totally	do not agree
9. My life feels meaningful right now.^1^	agree totally	do not agree
10. I have felt very stressed during the past year.^3^	do not agree	agree totally
11. I feel very stressed right now.^3^	not stressed	very stressed
*The following questions concern your situation during the past month*		
12. Do you feel fully recovered at awakening?*	daily	never
13. Do you have difficulties in falling asleep in the evening?^4^	never	daily
14. Do you have periods of awakening with difficulties in falling asleep again at night?^4^	never	daily
15. Do you feel physically exhausted?*	never	daily
16. What is your energy level?*	very high	very low
17. How is your overall satisfaction with your social life?^1^	satisfied	dissatisfied
18. How is your overall satisfaction with your home conditions?^1^	satisfied	dissatisfied
19. How is your overall satisfaction with your working conditions?*	satisfied	dissatisfied
*Have you had any trouble during the past month with:*		
20. Headache*	never	daily
21. Anxiety/restlessness*	never	daily
22. Depression^2^	never	daily
23. Restlessness^2^	never	daily
24. Irritability^2^	never	daily
25. Zest for life^2^	never	daily
26. Concentration difficulties^6^	never	daily
27. Abnormal forgetfulness^6^	never	daily
28. Heart symptoms (palpitations, irregular heart beats, pressure/pain in the chest)*	never	daily
29. Dizziness*	never	daily
30. Loss of appetite*	never	daily
31. Gastrointestinal problems (nausea, flatulence, acid regurgitation, pain/burn in the pit of the stomach)*	never	daily
32. Neck, shoulder, and/or back pain*	never	daily
33. Diffuse muscle pain*	never	daily
34. Problems with learning^6^	never	daily
35. Memory difficulties^6^	never	daily
36. I concentrate and take one step at a time when I handle strain.^8^	agree totally	do not agree at all
37. There is one or several persons that support me.*	agree totally	do not agree at all
38. I give more support to others than I get myself.*	do not agree at all	agree totally
39. I give myself the time I need for rest and relaxation.^7^	agree totally	do not agree at all
40. I give myself the time I need for stimulating leisure activities. ^7^	agree totally	do not agree at all
41. I prioritize recovery in my daily life.^7^	agree totally	do not agree at all
42. I can easily ‘charge my batteries’.^8^	agree totally	do not agree at all
43. I can easily mobilize extra energy when needed.^8^	agree totally	do not agree at all

1–8 refer to factor analysis grouping, see Material and methods;        Cronbach's alpha

^1^Feeling of meaningfulness with your home situation and social situation        0.897

^2^Mental symptoms                                         0.868

^3^Subjective level of stress                                     0.896

^4^Quality of sleep                                           0.872

^5^General health                                           0.897

^6^Difficulties with concentration and memory                          0.884

^7^Ability to prioritize recovery from stress                             0.812

^8^Ability to cope with stress                                     0.730

*= Not part of any of the factors above.
